# Network properties of control and epileptic human slice recordings

**DOI:** 10.1186/1471-2202-12-S1-P51

**Published:** 2011-07-18

**Authors:** Jennifer D Simonotto, Marcus Kaiser, Miles A Whittington, Mark O Cunningham

**Affiliations:** 1 School of Computing Science, Newcastle University, Newcastle upon Tyne, NE1 7RU, UK; 2 Institute of Neuroscience, Newcastle University, Newcastle upon Tyne, NE2 4HH, UK; 3 Department of Brain and Cognitive Sciences, Seoul National University, 599 Gwanak-ro, Gwanak-gu, Seoul 151-742, South Korea

## 

Of clinical interest are methods and tools to differentiate and localize epileptic foci, for surgical (and possibly other forms of intervention in the future) intervention. Previously, high frequency events, ripples (80-200 Hz) and fast ripples (200-500 Hz), were identified from both *in vitro* and *in vivo* recordings of epileptic foci tissue [1], but results were limited in that the data acquired was from single electrodes, and so simultaneous recordings of network activity were not obtained, and thus network dynamics within such regions were not analyzed. Using a 10 x10 grid Utah array electrodes for recording from focal epileptic and control slices of human tissue (control tissue being non-epileptic cortical tissue excised during extraction of a sub-cortical tumor or focus area), we are able to probe the network microstructure and, more critically, how these networks (epileptic focus and control) differ from each other. For analysis, we used standard low-frequency bands (theta, delta, alpha, gamma) as well as higher frequency bands (80-200 Hz, 200-500 Hz) for characteristic signals differentiating the epileptic and control populations. The raw data was filtered at these separate bands and networks were extracted from the filtered data using phase synchronization and cross-correlation as a means to detect phase and amplitude signal similarities, respectively. Once the networks were extracted, we could examine different network measures to determine if network properties were statistically different or not. We measured small-worldness characteristics such as the Clustering Coefficient (which measures how similar a reference node’s neighbour’s connectivity are to the reference node), Characteristic Path Length (which measures how many connections it takes on average to get from any one node to any other node in the network), degree (number of connections each node has), % change of network volatility, Small Worldness coefficient (Normalized Clustering Coefficient/Normalized Characteristic Path Length), modularity Q, and statistical information such as the number and size of (unthresholded) network clusters were extracted and compared across datasets and population.
